# Characterization of a Novel Porcine Parvovirus Tentatively Designated PPV5

**DOI:** 10.1371/journal.pone.0065312

**Published:** 2013-06-07

**Authors:** Chao-Ting Xiao, Luis G. Giménez-Lirola, Yong-Hou Jiang, Patrick G. Halbur, Tanja Opriessnig

**Affiliations:** Department of Veterinary Diagnostic and Production Animal Medicine, Iowa State University, Ames, Iowa, United States of America; Virginia Polytechnic Institute and State University, United States of America

## Abstract

A new porcine parvovirus (PPV), provisionally designated as PPV5, was identified in U.S. pigs. Cloning and sequencing from a circular or head-to-tail concatemeric array revealed that the PPV5 possesses the typical genomic organization of parvoviruses with two major predicted open reading frames (ORF1 and ORF2), and is most closely related to PPV4 with overall genomic identities of 64.1–67.3%. The amino acid identities between PPV5 and PPV4 were 84.6%–85.1% for ORF1 and 54.0%–54.3% for ORF2. Unlike PPV4, but similar to bovine parvovirus 2 (BPV2), PPV5 lacks the additional ORF3 and has a much longer ORF2. Moreover, the amino acid sequences of ORF1 and ORF2 of BPV2 showed higher homologies to PPV5 than to PPV4. The conserved motifs of the Ca^2+^ binding loop (YXGXG) and the catalytic center (HDXXY) of phospholipase A_2_ (PLA_2_) were identified in VP1 (ORF2) of PPV5, as well as in BPV2, but were not present in PPV4. Phylogenetic analyses revealed that PPV5, PPV4 and BPV2 form a separate clade different from the genera *Parvovirus* and *Bocavirus*. Further epidemiologic investigations of PPV4 and PPV5 in U.S. pigs of different ages indicated a slightly higher prevalence for PPV5 (6.6%; 32/483) compared to PPV4 (4.1%; 20/483), with detection of concurrent PPV4 and PPV5 in 15.6% (7/45) of lungs of infected pigs. Evidence for potential vertical transmission or association with reproductive failure was minimal for both PPV4 and PPV5. The high similarity to PPV4 and the lack of ORF3 may suggest PPV5 is an intermediate of PPV4 during the evolution of parvoviruses in pigs.

## Introduction

Parvoviruses are ubiquitous and are associated with a broad spectrum of clinical diseases in animals, including reproductive failure, enteritis, panleukopenia, hepatitis, erythrocyte aplasia, immune complex-mediated vasculitis, and cerebellar ataxia [1], [2]. The family *Parvoviridae* is composed of two subfamilies: *Parvovirinae*, infecting vertebrates, and *Densovirinae*, infecting arthropods. The subfamily *Parvovirinae* can be further divided into five genera: *Parvovirus*, *Erythrovirus*, *Dependovirus*, *Amdovirus* and *Bocavirus* [2].

Parvoviruses are small, non-enveloped, single-stranded DNA viruses, with a genome size of approximately 4–6.3 kb that contain terminal palindromic sequences [2]. In general, there are two major open reading frames (ORFs) encoding for the non-structural protein(s) located at the 5′-end and the capsid protein(s) located at the 3′-end. An additional ORF3 is located in the middle of the viral genome among *Bocavirus* members [2], [3]. With the recent advent and use of better molecular assays and pathogen discovery tools, several novel members of the subfamily *Parvovirinae* have been discovered [4]–[15].

Five different groups of parvoviruses that infect pigs have been identified to date, including classic porcine parvovirus (PPV) type 1 (PPV1), PPV2, PPV3 (also known as porcine PARV4, hokovirus, or partetravirus), PPV4, and porcine bocaviruses (PBoV). All of these viruses are genetically divergent from each other [2], [4], [6], [8], [13], [14], [16], [17]. The overall prevalence of the recently identified parvoviruses in pig herds varied from 6.4% to 20% for PPV2, 9.7% to 12.4% for PPV3, and 1.5% to 39.7% for PBoV [5], [18]–[21].

PPV4 was initially identified in 2010 in U.S. pigs diagnosed with porcine circovirus (PCV) associated disease, and subsequently was also identified in China, Hungary, and Africa [6], [22], [23]. PPV4 is unique in that its genome nucleotide sequence is most closely related to bovine parvovirus 2 (BPV2), but its genome organization, characterized by presence of an additional ORF3, resembles that of *Bocavirus* [6]. Further characterization revealed that PPV4 had a circular or a head-to-tail concatemeric template in its DNA [6], which is different from the traditional head-to-head or tail-to-tail intermediates, but similar to those of adeno-associated virus (AAV) and bocaviruses of different species [24]–[27]. The amino acid identities between PPV4 and the closest BPV2 were 33.6% for ORF1 and 24.5% for ORF2, while the amino acid identities between PPV4 and bocaviruses have been described to range from 4.9–11.2% for ORF3 [6]. The prevalence rate of PPV in Chinese swine herds was determined to be 2.1% in clinical samples and 0.8% in samples from healthy animals [22]. A prevalence rate of 6.4% was detected in pigs in Hungary [19].

During an initial investigation of the PPV4 prevalence in U.S. pigs, a novel PPV, provisionally named PPV5, was identified [28] and showed the closest relationship (overall genome sequence identities of 64.1–67.3%) to PPV4. Detailed genomic characterization at the nucleotide and amino acid levels during the present study revealed that the PPV5 possesses two predicated ORFs, similar to BPV2 but different from PPV4, and that the conserved motifs of the Ca^2+^ binding loop (YXGXG) and the HDXXY in the catalytic center of phospholipase A_2_ (PLA_2_) are present in the capsid protein (VP1) of PPV5 but are lacking in PPV4. Moreover, PPV5 has a slightly higher prevalence than PPV4 in U.S. pigs. In addition, antibodies against PPV1 do not react with PPV4 and PPV5 capsid proteins perhaps supporting that development of differential serological tools would be feasible and could be utilized in the future to further characterize the infection dynamics of emerging parvoviruses such as PPV4 and PPV5.

## Materials and Methods

### Ethics Statement

All samples utilized were arbitrarily selected and originated from sick pig case submissions to the Iowa State University Veterinary Diagnostic Laboratory (ISU-VDL) for diagnostic work-up. The sample collection and submission was unrelated to and not part of this study. The protocol for this study was approved by the Iowa State University Institutional Biosafety Committee (permit number 12-D/I-0038-A).

### Clinical Samples

A total of 483 lung samples and 154 fecal samples were obtained from pigs that originated on 263 farms in 18 U.S. states including Colorado, Iowa, Illinois, Indiana, Kansas, Michigan, Minnesota, Missouri, North Carolina, North Dakota, Nebraska, Ohio, Oklahoma, Pennsylvania, Texas, Virginia, Wisconsin, and Wyoming (Tables 1 and 2). The pigs suffered from a variety of clinical signs such as respiratory disease, diarrhea, systemic disease, or reproductive disorders. The age of the pigs sampled ranged from neonatal to adult. In addition, 184 samples (39 fetal thoracic fluids and 6 serum samples from aborted fetuses, 61 serum samples from healthy pre-suckle piglets, and 78 serum samples from healthy post-suckle piglets) from 39 farms located in Iowa, Illinois, Minnesota, Nebraska and North Carolina were utilized.

**Table 1:** Summary of the history (age, states, farms) and prevalence of PPV4, PPV5 or concurrent PPV4/5 DNA in lung samples obtained from 483 pigs located in the U.S.

Age group	No. of farms/states	No. of samples	PPV4	PPV5	Concurrent PPV4/5
Fetuses(<1 day)	11/7	28	0/28 (0%)	0/28 (0%)	0/28(0%)
Suckling pigs (1–20 days)	7/3	15	0/15 (0%)	0/15 (0%)	0/15 (0%)
Nursery pigs (21–55 days)	75/14	178	2/178 (1.1%)	2/178 (1.1%)	1/178 (0.5%)
Grow-finish pigs (8–25 weeks)	108/14	235	18/235 (7.7%)	29/235 (12.3%)	6/235 (2.6%)
Mature pigs (>25 weeks)	20/7	27	0/27 (0%)	1/27 (3.7%)	0/27 (0%)
**Total**	**183/17**	**483**	**20/483 (4.1%)**	**32/483 (6.6%)**	**7/483 (1.4%)**

**Table 2:** Summary of the history (age, states, farms) and prevalence of PPV4, PPV5 or concurrent PPV4/5 DNA in 185 fecal samples from pigs in the U.S.

Age group	No. of farms/states	No. of samples	PPV4	PPV5	Concurrent PPV4/PPV5
Suckling pigs (1–20 days)	17/6	33	0/33 (0%)	1/33 (3%)	0/33 (0%)
Nursery pigs (21–55 days)	46/9	59	1/59 (1.8%)	0/59 (0/%)	0/59 (0/%)
Grow-finish pigs (8–25 weeks)	32/10	56	2/56 (3.6%)	3/56 (5.4%)	0/36(0%)
Mature pigs (>25 weeks)	5/3	6	0/6 (0%)	0/6 (0%)	0/6 (0%)
**Total**	**80/15**	**154**	**3/154 (1.9%)**	**4/154 (2.6%)**	**0/154 (0%)**

The tissue samples of approximately 1 gram were minced and diluted 1∶10 in Dulbecco’s Modified Eagle Medium (DMEM), homogenized by using a Stomacher® 80 (Seward Laboratory Systems Inc, Bohemia, NY), and centrifuged at 1,500×*g* for 10 min to obtain supernatant. Fecal samples of approximately 0.4 gram were resuspended 1∶10 (weight/volume) in phosphate-buffered saline (PBS), vortexed for 30 sec, and centrifuged at 1,500×*g* for 10 min. All samples were stored at −80°C until usage.

To evaluate potential cross-reactivity between PPV1, PPV4 and PPV5 capsid proteins the following samples from nursery pigs with known PPV1 status, determined by a hemagglutination inhibition (HI) assay [29], were utilized: 18 experimental samples with 6/18 negative control samples, 3/18 samples from pigs with low levels of passively derived antibodies, and 9/18 positive control samples collected 21 days post experimental infection with PPV1 as described [30], [31]. In addition to samples from experimentally infected pigs, 22 field samples from breeding sows with known positive HI status from two different farms were tested.

### Viral DNA Extraction

DNA was extracted from 50 µl of tissue homogenates, fecal suspensions, serum samples, or fetal thoracic fluids, using the 5×MagMAX™ 96 Viral Isolation Kit (Ambion) according to the manufacturers’ instructions on an automated extraction platform (KingFisher Flex; Thermo Fisher Scientific Inc). DNA was eluted in 50 µl of elution buffer provided in the kit.

### Cloning and Genome Sequencing

The known genomic sequences of PPV4 were downloaded from the GenBank and analyzed with the Lasergene package (DNASTAR Inc.). A pair of primers (PPV4F/PPV4R), located in the conserved region of the replicase gene, was designed to amplify a 704 bp fragment of PPV4 (Table 3). The PCR products were separated on a 1% agarose gel by gel electrophoresis, the target bands were excised and purified with the QIAquick® gel extraction kit (Qiagen), and cloned into the pCR®II-TOPO® vector (Invitrogen). The recombinant plasmids were transformed into TOP10 *Escherichia coli* bacteria (Invitrogen) and propagated following the procedures of the cloning kit manual. The identified recombined plasmids were extracted using the QIAprep Spin MiniPrep kit (Qiagen) according to the manufacturers’ instructions, quantified using a spectrophotometer (Nanophotometer, IMPLEN), and then sequenced.

**Table 3:** Primers and probes used for PPV4 and PPV5 detection.

Primers/probes	Sequence (5′-3′)	Position
PPV4F	GCATTGGTGTGTGTCTGTGTCC	1068–1089^a^
PPV4R	TCCTCTTTCCCGTTTGTTTCAT	1750–1771^a^
PPV4DF	GCATTGGTGTGTGTCTGTGTCC	1068–1089^a^
PPV4DR	GTGGCACATTTGTACATGGGAG	1391–1412^a^
PPV4-Probe	FAM-5′- CTCCGCGGGATGTGCTTACAATTTTCA -3′-BHQ	1097–1123^a^
PPV5-Probe	CAL Fluor Orange 560-5′- ACTTTGGTGTTGAGGGACTTAGCTTTTTTGTAC -3′-BHQ	1292–1324^b^

a Nucleotide position according to PPV4 (GenBank accession no. GQ387500).

b Nucleotide position according to PPV5 (GenBank accession no. JX896321).

The majority of the genome sequence of the newly identified parvovirus was obtained by sequence-independent single primer amplification (SISPA) as described previously [32], [33], with some modification. Briefly, a new complementary strand was generated by incubating 20 µl of the extracted DNA with 1 µl (5 units) of Klenow fragment (3′->5′ exo^-^) (New England Biolabs) and 5 pmol of primer PPV5RN (5′-GGTACGCACTCGTCGTCTAGTAGTTNNNNNNNN-3′) at 60°C for 10 s, followed by an additional incubation at 37°C for 1 h. Five of the obtained products were used as templates in a subsequent PCR reaction, in which a method similar to primer walking was adopted. In each reaction the same reverse primer PPV5R (5′- GGTACGCACTCGTCGTCTAGTAGTT-3′) was used, with different walking forward primers designed initially based on the known PPV5 fragment of 704 bp and subsequently based on the sequences obtained after each round of amplification. The obtained PCR products were then cloned and sequenced as described above. As previously described to amplify the genome ends of PPV4 [6], [22], the genome termini of PPV5 were determined by inverse PCR. Specifically, the circular or head-to-tail concatemeric templates were amplified with the orientation of forward and reverse primers contrary to the normal PCR for linear genome amplification. New sequencing primers were designed based on the first PPV5 genome obtained. All primers used for the genome sequencing are listed in Table 4. These primers were utilized to amplify the whole PPV5 genome for several more PCR positive samples to confirm the presence of PPV5 DNA in different geographic locations.

**Table 4:** Primers used for the PPV5 genome sequencing.

Primers	Sequence (5′–3′)
PPV5U1F	CAAACATGGAGCGGGAGAAGAC
PPV5U2F	GCCAGGACATCGCTACACAGGT
PPV5U3F	GCTAACAGTGCCTGGTCATCTTC
PPV5U4F	TGGTCATCTTCCAGGACAAACTTAT
PPV5U5F	CCAACAAAGTGACATGGAGTGCTC
PV5-U6F	CCTCTGCTTGGCAAACGCTT
PV5-U7F	CTAACAGTGCCTGGTCATCTTC
PPV5RN	GGTACGCACTCGTCGTCTAGTAGTTNNNNNNNN
PPV5R	GGTACGCACTCGTCGTCTAGTAGTT
PPV5R1537	GTGGCACATTTGTACATGGGAG
PV5-1017R	CGCCAGACTCACAGTTTTCATT
PV5-386R	GCCAAATATCTCGTCGGTCTGT
PPV5F108	GGGATGTGACGCAGTACAGACC
PV5R1330	CCAACCACTGTACAATGTCTAGCAT
PV5F1094	GGAAAAGCACAGATGAAGGGTT-
PV5R3651	GTAAGGATGTCTGTGGAGGATCAAT
PV5F3147	GAACACGACTCAAGCGAAGAAC
PV5R4625	GACAATCCAGGGAACGAGGTAAC
PV5F4180	GTTTTGAGGATACACATTACGAGC
PV5R5553	AAAAAAAGAGGAAGAACTATTTTTTTCTC
PV5R311	ATAAATTATGCAAAGTAGGAGGAGTC
PV5F5364	CATACTCCGAGGTGGAACCCT
PV5R422	ATCAGGTTAACTTCCTCTTTGCAT
PV5F5346	GTTGTGAGGCGAAAGAAACATACT

### Sequence and Phylogenetic Analysis

The sequences were assembled and analyzed with the software DNASTAR (Lasergene®) and DNAMAN Version 7 (Lynnon Corporation). Sequences were aligned by ClustalW and phylogenetic analyses were carried out with MEGA 5.0 [34]. The evolutionary trees were constructed by the Maximum Likelihood method based on the nucleotide sequences of non-structural gene (ORF1) and capsid gene (ORF2), with Kimura 2-parameter model and bootstrap test of 1000 replicates. For the amino acid sequences of the non-structural protein (NS1, ORF1) and the capsid protein (VP1, ORF2) the Poisson correction model and bootstrap test of 500 replicates were utilized [34].

### Development of a Duplex Real-time PCR Assay to Investigate the Prevalence of PPV4 and PPV5

Based on the known sequences of PPV4 and PPV5 obtained in the present study (the sequencing results of PCR products based on PPV4F/PPV4R, 704 bp), a pair of detection primers (PPV4DF/PPV4DR) and two TaqMan® probes specific for PPV4 and PPV5 were designed, respectively, with hairpin and dimers within primers and probes tested by Primer Express software (Version 3.0; Applied Biosystems). The probes were labeled with 5′-carboxyfluorescein (FAM) or CAL Fluor® Orange 560 at the 5′-end and Black Hole Quencher™ (BHQ) at the 3′-end. Primer and probe sequences are shown in Table 3.

The real-time PCR reactions were carried out in 96-well plates. Standards were run in triplicate, and each reaction consisted of a total volume of 25 µl, containing 12.5 µl of TaqMan® Universal PCR Master Mix (Applied Biosystems), 2.5 µl of sample or standard DNA, 1 µl of 10 µM of each of the two primers, 0.5 µl of each of the two 10 µM probes, and 7 µl distilled water. Amplification and quantification were performed using the ABI 7500 Fast Real Time PCR System (Applied Biosystems) under universal conditions: 2 min at 50°C 10 min at 95°C, 40 cycles of 15 s at 95°C and 1 min at 60°C. The sensitivity of the real-time PCR was determined by using serial dilutions of the plasmids obtained above as standards according to a method described previously [35]. The specificity of the probes was confirmed by BLAST analysis, and by using DNA samples positive for other DNA viruses including PPV1, PPV2 [21], PPV3 [20], PCV type 1 (PCV1), PCV2, Torque teno sus virus 1 (TTSuV1), and TTSuV2 [35].

### Protein Expression, Purification and Enzyme-linked Immunosorbent Assay (ELISA) Development

Based on the predicted antigenic sites as determined by analysis with DNASTAR, one pair of primers was designed to amplify the partial capsid gene of each PPV1, PPV4 and PPV5. The following primers were utilized: PPV1F 5-CGGGATCCCTAATGGTCGCACTAGACAC, PPV1R 5- GCGTCGACATGCATGTTAGATTTCCCT-3; PPV4F 5-CGGGATCCGAATATCTAAAAAACAT -3, PPV4R 5-TAGTCGACAATGGATAGTGGTGGTG-3; PPV5F 5-CCGGATCCGAAAACATTGATAACAT-3, and PPV5R 5-TAGTCGACAATAGATAAAGGGGGCG-3, with restriction sites for *Bam*H I within the forward primers and for *Sal* I within the reverse primers (indicated by underlining), which correspond to proteins of 267 amino acids for PPV1, 106 amino acids for PPV4, and 96 amino acids for PPV5. The gene fragments were amplified, expressed and identified according to methods described previously [36] except for using the expression vector pHUE (a gift from Dr. R. Rowland, Kansas State University). The proteins were then purified with PrepEase® His-tagged Protein Purification Midi Kit-High Specificity (USB Corp. Germany) according to the manufactures’ instructions and evaluated in an ELISA platform [36] for cross-reactivity between PPV1, PPV4 and PPV5 antibodies. Briefly, microtiter plates were coated with each of the three recombinant polypeptides, diluted in phosphate-buffered saline (PBS) at a concentration of 1 µg/ml each and incubated overnight at room temperature. Plates were blocked with a fetal bovine serum solution, washed, and then incubated with sera diluted 1∶100 in PBS containing fetal bovine serum for 60 min at 37°C. After a wash step, a 1∶10.000 dilution of peroxidase-conjugated goat anti-swine immunoglobulin G (IgG) (Jackson) was added and incubated at 37°C for 60 min. Finally, the peroxidase reaction was visualized by using a tetramethylbenzidine-hydrogen peroxide solution as a substrate (KPL).

### Nucleotide Sequence GenBank Accession Numbers

The genome sequences of PPV5 obtained in the present study have been deposited into the GenBank data base under the accession numbers JX896318 through JX896322.

## Results

### Identification and Genomic Characterization of PPV5

During the initial screening of the tissue samples by conventional PCR with primers PPV4F/4R, several PCR positive samples were detected. Four of the PCR products were purified, cloned and sequenced. The obtained sequences all had a length of 704 nucleotides, and these sequences were compared with nine published PPV4 sequences, which included two from the U.S. (GQ387499 and GQ387500) and seven from China (GU978965, GU978964, GU978966, GU978967, GU978968, HM031135 and HM031134). Notably, three of the four investigated PCR products displayed only about 76% nucleotide identity to the known PPV4 sequences, indicating a possible new PPV4 subtype or a new parvovirus closely related to PPV4. The other sequence had a high identity (around 99%) to the published PPV4 sequences. Subsequently, the whole genome of PPV5 strain ND564 from North Dakota was obtained [28] with the methods of SISPA and inverse PCR. Moreover, three other whole PPV5 genomes (strain MI216 from Michigan, strain IN273 from Indiana and strain IA469 from Iowa) were further obtained with the primers designed based on the sequence of ND564. Among them, PPV5-IA469 was revealed to have two variants with different genome sizes (5,805 bp and 5,756 bp) likely because of differences in the length of the repeated sequences in the euntranslated regions (UTRs) similar to PPV4 reported previously [6], [22].

**Figure 1:**
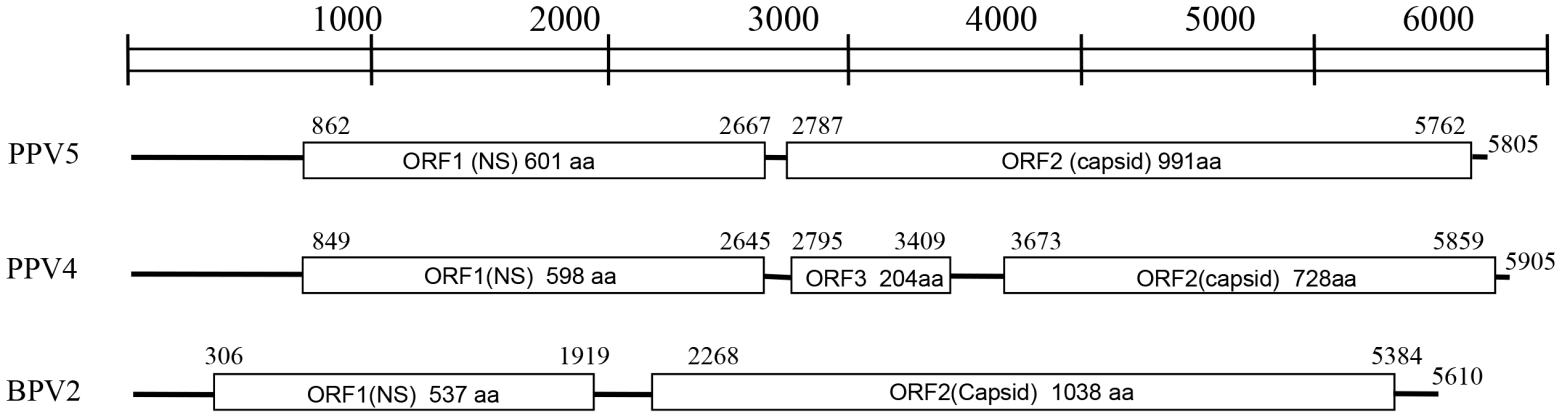
Schematic representation of the genomes of PPV5 compared with PPV4 and BPV2. The position and size of putative ORFs are indicated. The GenBank accession numbers of the reference sequences are JX896321 (PPV5), GQ387499 (PPV4), AF406966 (BPV2). aa: amino acid. NS: nonstructural gene.

**Figure 2:**
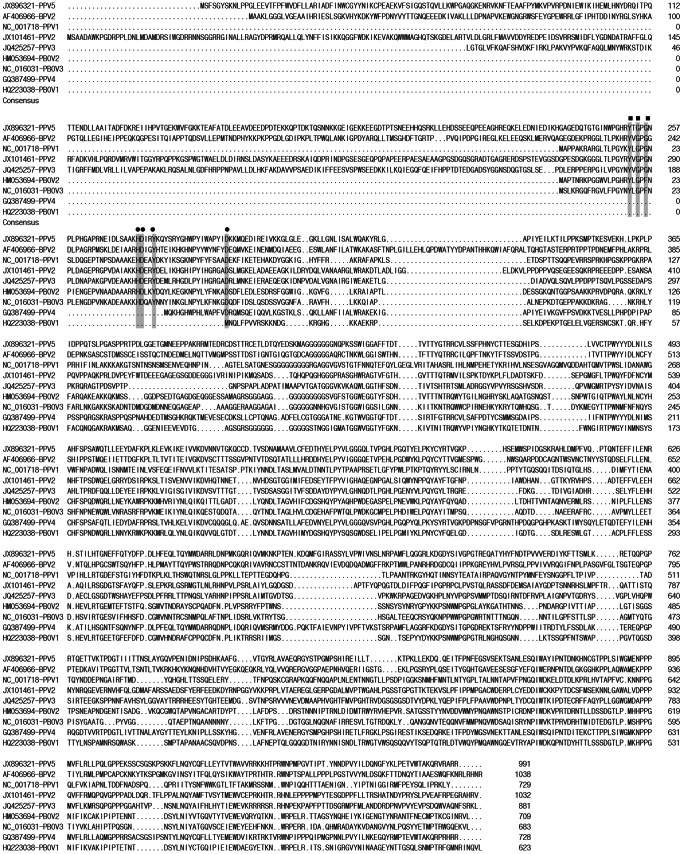
Amino acid sequence alignment of VP1 with the putative phospholipase A_2_ motif of PPV5, other PPVs, PBoV, and BPV2. The Ca^2+^ binding loop is indicated by filled squares and the catalytic residues are indicated by filled circles. “.” indicates a deletion compared to the strain on top. The positions of the amino acids and the GenBank numbers of the sequences are indicated.

The genome sizes of PPV5 obtained in the present study varied from 5,516 bp to 5,805 bp, with typical PPV genome organization of two predicted major ORFs, ORF1 and ORF2, and 5′ and 3′ UTRs. However, the ends of the genomes were arbitrarily assigned, as the left and right ends of the linear viral genome were not determinable from the head-to-tail or circular configuration obtained by inverse PCR, as described for PPV4 [6] (Figure 1). The ORF1 encodes a putative NS1 of 601 amino acids and ORF2 encodes a putative VP1 of 991 amino acids. The overall genome sequence identities within PPV5 strains were 97.1%–99.5%, while the PPV5 genomes showed rather low identities of 64.1%–67.3% to the known PPV4 genomic sequences. Specifically, the nucleotide and amino acid sequence identities of ORF1 were 99.2%–100% and 98.7%–100% among PPV5 strains, and were 77.4%–78.2% and 84.6%–85.1% between PPV5 and PPV4 strains, respectively. Moreover, the nucleotide and amino acid sequences of ORF2 showed identities of 99.2%–100% and 99.5%–100% within the PPV5 strains, while they showed identities of 59.2%–60.1% and 54.0%–54.3% between PPV5 and PPV4 strains, respectively. Interestingly, the extra ORF3 (204 amino acids, GQ387499) described for the PPV4 genome was not present in the PPV5 genome, but with 991 amino acids in length, ORF2 of PPV5 was 263 amino acids longer compared to ORF2 of PPV4, which is only 728 amino acids in length. The additional portion of the ORF2 in PPV5 displayed only 11% similarity to PPV4 ORF3, and no putative conserved domains or significant similarity were detected by protein BLAST. Additionally, the VP1 protein of PPV5 (JX896321) showed identities of 14.8% with PPV1 (NC_001718), 20.6% with PPV2 (JX101461), 26.9% with PPV3 (JQ425257), 19.4% with PBoV1 (HQ223038), 16.6% with PBoV2 (HM053694), and 11.3% with PBoV3 (NC_016031) indicating great divergence within the capsid protein. The alignments of the amino acid sequences of VP1 of PPV1-PPV5, PBoV1-PBoV3 and BPV2 are shown in Figure 2. As a result of the deletion and insertion of repeat sequences similar to the PPV4 genome [6], [22], the length of the UTR varied from 615 bp to 904 bp for PPV5, albeit the mechanism or function of the sequence deletion/insertion is currently unknown.

In contrast to PPV4, the genomic organization of PPV5 was found to be more similar to BPV2 (AF406966) which also possesses two large ORFs, ORF1 encoding a protein of 537 amino acids and ORF2 encoding a protein of 1,038 amino acids. Moreover, the amino acid identities between PPV5 and BPV2 were 38.6%–38.8% for ORF1 and 28.3% for ORF2, while similarities between PPV4 and BPV2 were only 33.6% for ORF1 and 24.5% for ORF2 [6], indicating a closer relationship of PPV5 with BPV2 than PPV4 with BPV2. Overall, PPV5 showed rather low amino acid identities of 17.3%–26.8% for ORF1 and 9.4%–27.4% for ORF2 to other members within the subfamily *Parvovirinae.*

According to the species demarcation criteria in the genus within *Parvovirinae* defined by the International Committee on Taxonomy of Viruses (ICTV) [2], we feel that PPV5 can be considered as a separate parvovirus species different from PPV4 based on it being <95% related by non-structural gene DNA sequence.

### Identification of a Phospholipase A_2_ (PLA_2_) Motif in the VP1 Protein of PPV5

**Figure 3:**
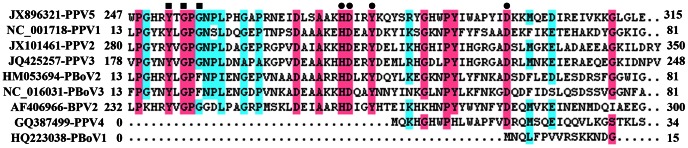
Sequence alignment of the putative phospholipase A_2_ motif of PPV5 with those of other PPVs, PBoV, and BPV2. The Ca^2+^ binding loop is indicated by filled squares and the catalytic residues by filled circles. The positions of the amino acids and the GenBank numbers of the sequences are indicated.

In the alignments of the predicted VP1 protein sequence of PPV5 with those of BPV2 and other parvoviruses in pigs, including PPV1, PPV2, PPV3, PPV4 and PBoVs, the conserved motifs of Ca^2+^ binding loop (YXGXG) and the catalytic residues (HDXXY) of PLA_2_ were identified in PPV5, including the conserved residue D63, which was reported to be required for parvovirus entry and infectivity (Figures 2 and 3) [37]–[39]. Interestingly, this PLA_2_ motif was also identified in BPV2, PPV1, PPV2, PPV3, PBoV2 and PBoV3, but not in PBoV1 and PPV4 (Figure 3). The length of VP1 of PPV4 is shorter than that of PPV5 with an extra ORF3; however, the enzyme domain was also not identified in the ORF3 of PPV4 (data not shown).

### Phylogenetic Analysis of PPV5

**Figure 4:**
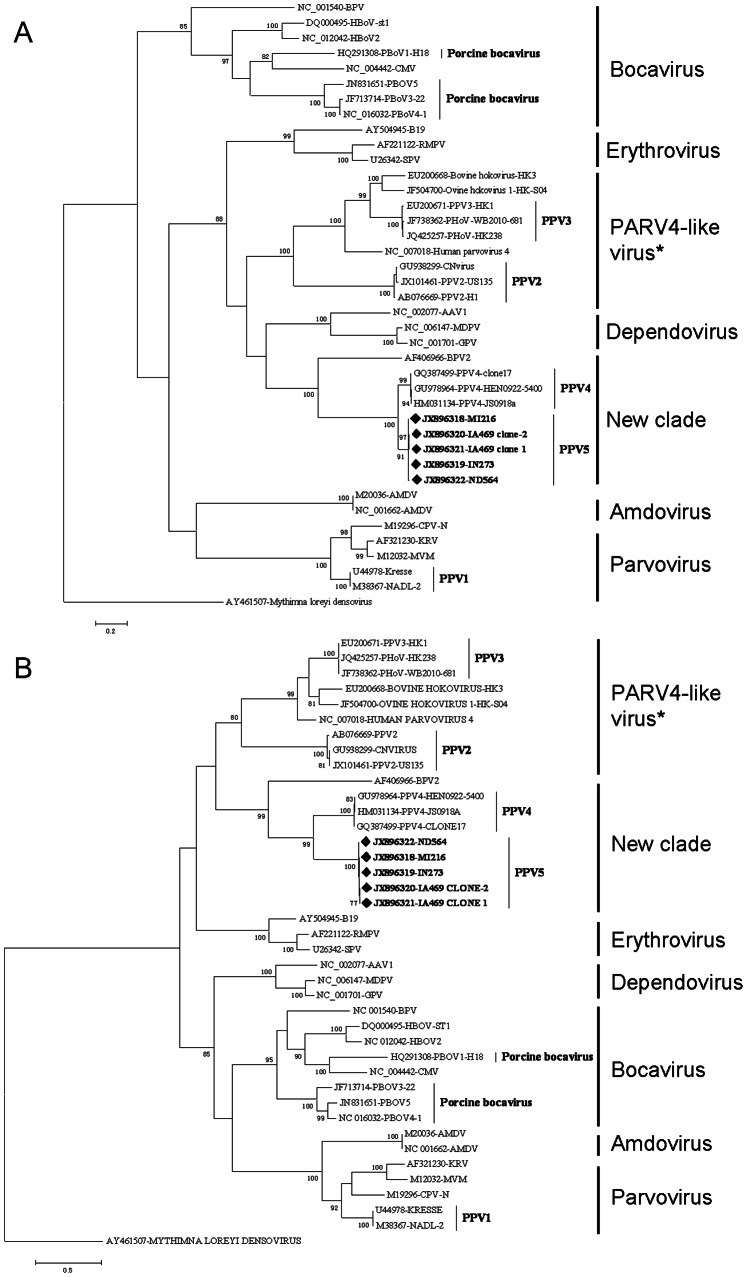
The phylogenetic trees were constructed by using the Maximum Likelihood method based on the Poisson correction model, with the amino acid sequences of the nonstructural protein (ORF1, NS1) and the capsid protein (ORF2, VP1). The percentage of the tree in which the associated sequences clustered together is shown above the branches (only >70% are shown). The trees are drawn to scale, with branch lengths measured in the number of substitutions per site. Evolutionary analyses were conducted in MEGA5 [34]. The analyses involved 39 sequences of vertebrate parvoviruses with their GenBank accession numbers marked in the tree. PPV5 sequences characterized in the present study are in bold and marked with “♦”. The sequence of *Mythimna loreyi* densovirus (AY461507) was used as outgroup to root the tree. The asterisk indicates the new genus proposed by ICTV. (A) Tree constructed based on the amino acid sequences of NS1. All positions containing gaps and missing data except ambiguous positions were included. There were a total of 900 positions in the final dataset. (B) Tree constructed based on the amino acid sequences of VP1. All positions containing gaps and missing data except ambiguous positions were included. There were a total of 1,177 positions in the final dataset.

**Figure 5:**
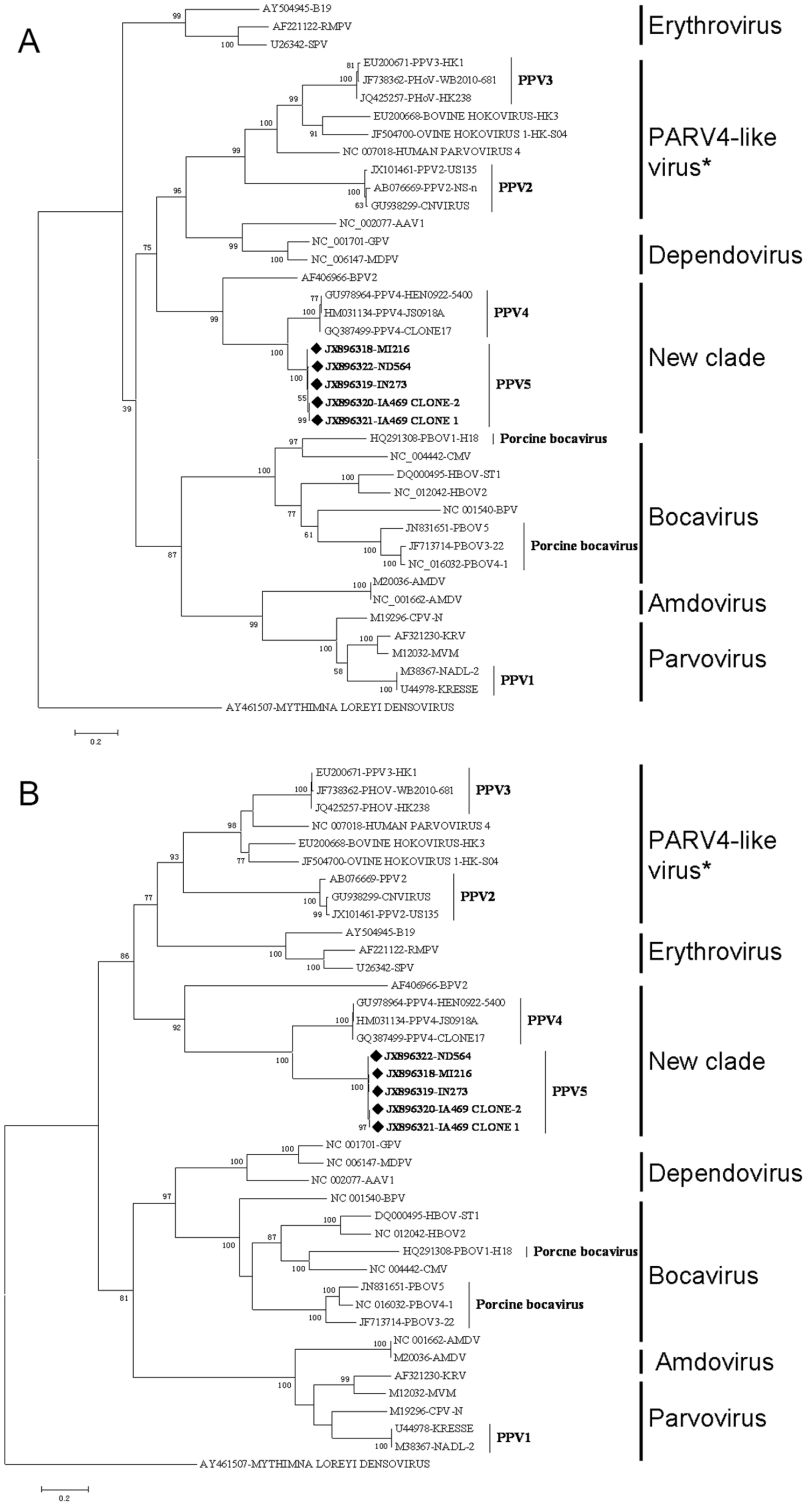
The phylogenetic trees were constructed by using the Maximum Likelihood method based on the Kimura 2-parameter model, with the nucleotide sequences the nonstructural protein (ORF1, NS1) and the capsid protein (ORF2, VP1). The percentage of the tree in which the associated sequences clustered together is shown above the branches (only those >70% are shown). The trees are drawn to scale, with branch lengths measured in the number of substitutions per site. Evolutionary analyses were conducted in MEGA5. The analyses involved 39 sequences of vertebrate parvovirus with their GenBank accession numbers marked in the tree. PPV5 sequences characterized in the present study are in bold and marked with “♦”. The sequence of *Mythimna loreyi* densovirus (AY461507) is used as outgroup to root the tree. The asterisk indicates the new genus proposed by ICTV. (A) Tree constructed based on the nucleotide sequence of NS1. All positions containing gaps and missing data except ambiguous positions were included. There were a total of 2,690 positions in the final dataset. (B) Tree constructed based on the nucleotide sequence of VP1. All positions containing gaps and missing data except ambiguous positions were included. There were a total of 3,386 positions in the final dataset.

To reveal the phylogenetic relationship of PPV5 with other parvoviruses, evolutionary trees were constructed based on the nucleotide and amino acid sequences of ORF1 and ORF2 of PPV5 identified in the present study and representative parvovirus sequences belonging to the subfamily of *Parvovirinae*. Evolutionary trees constructed based on nucleotide and amino acid sequences of NS1 and VP1 had similar topology. The trees based on the amino acid sequence of NS1 and VP1 are shown in Figure 4 and the nucleotide-based trees are shown in Figure 5. PPV5 strains have the closest relationship with PPV4 strains, but form a separate subclade distinct from PPV4 in all of the four trees, supported by bootstrap tests. Moreover, PPV5 also shows a close relationship to BPV2, indicating that PPV5, PPV4 and BPV2 could be classified into an independent genus within subfamily *Parvovirinae*.

### Validation of the Duplex Real-time PCR Assay

Ten-fold serial dilutions of the obtained plasmids pCRII-PPV4 and pCRII-PPV5 were used to construct standard curves by plotting the logarithm of the plasmid copy number against the measured cycle threshold (C_T_) values. The average slopes, R2 and intercept values of the standard curves were −4.001, 0.997 and 46.270 for PPV4 and −3.894, 0.992 and 46.178 for PPV5. The specificity of the duplex real-time PCR was tested by BLAST analysis of the probes and by testing available pig virus DNA including PPV1, PPV2, PPV3, PCV1, PCV2, TTSuV1, and TTSuV2 with no identified cross-amplification. Quantitative analysis identified the detection limit of viral DNA with 50 genome equivalents, occurring around cycle 39 for both PPV4 and PPV5 and for all replicates. This demonstrated that the duplex real-time PCR assay is valid for detection of both PPV4 and PPV5.

### Prevalence of PPV4 and PPV5 DNA in the U.S

The combined prevalence of PPV4 and PPV5 in lung tissues was 9.3% (45/483). Specifically, the prevalence was 1.7% (3/178) for nursery pigs, 17.4% (41/235) for grow-finish pigs and 3.7% (1/27) for mature pigs. The detailed results are summarized in Table 1. Lung homogenates positive for PPV5 and PPV4 had C_T_ values of 28–38.9 and 29.5–39 and the amount of PPV5 and PPV4 DNA ranged from 3.0×10^4^ to 1.8×10^7^ and 2.6×10^4^ to 6.2×10^6^ genomic copies per ml of the homogenate, respectively.

The combined prevalence of PPV4 and PPV5 in fecal samples was 4.5% (7/154). Specifically, the prevalence was 3% (1/33) for suckling pigs, 1.7% (1/59) for nursery pigs, and 8.9% (5/56) for grow-finish pigs. The detailed results are summarized in Table 2. Fecal samples positive for PPV4 and PPV5 had C_T_ values of 32.7–39.4 and 36.1–39.7, and the amount of PPV4 and PPV5 DNA ranged from 2.0×10^4^ to 9.8×10^5^ and 1.9×10^4^ to 1.5×10^5^ genomic copies per ml fecal supernatant, respectively.

For sera or thoracic fluids, 0.5% (1/184) of the samples were positive for PPV5 and 4.9% (9/184) were positive for PPV4. Specifically, 17.9% (7/39) of fetal thoracic fluids from five different farms were PPV4 PCR positive, with high C_T_ values of 35.3–39.2 and the amount of PPV4 DNA ranged from 2.4×10^4^ to 1.8×10^5^ genomic copies per ml fecal thoracic fluid. The low detection rates for PPV5 appear to not support an association of PPV5 with abortion based on the limited sample set evaluated. In addition, although PPV4 was detected in 17.9% of fetal thoracic fluids, the viral loads were low and there were only 1/61 pre-suckling and 1/78 suckling serum samples positive for PPV4, and 1/78 suckling pig serum samples was positive for PPV5. Together with the epidemiologic results from lung and fecal samples described above, this likely suggests that vertical transmission for both PPV4 and PPV5 is minimal.

Co-infection of PPV4 and PPV5 was observed in 15.6% (7/45) of positive lung samples but was not detected in fecal samples, serum samples, or thoracic fluids.

### Evaluation of Cross-reactivity of the Partial Capid Protein of PPV1, PPV4 and PPV5

Among the experimentally derived samples, all six negative control samples were negative by the PPV1 ELISA and the PPV4 ELISA while 1/6 samples were positive by the PPV5 ELISA. The three samples from pigs with passively derived antibodies were negative by the PPV1 ELISA and the PPV5 ELISA but one sample was positive for PPV4. Finally, among the positive controls, 9/9 were positive for PPV1 and 0/9 were positive for PPV4 and PPV5. Among the 22 field samples, 17/22 were positive for PPV1, 4/22 were positive for PPV4 (all four samples were also positive for PPV1) and 3/22 samples were positive for PPV5 (all three samples were also positive for PPV1 and PPV4). Of note, 3/22 field samples were positive for all three PPVs. These preliminary results indicate that the PPV1 ELISA appears to correlate well with the hemagglutination inhibition assay and that cross reaction with PPV4 and PPV5 is unlikely. However, testing of additional samples including known positive samples from pigs infected with either PPV4 or PPV5 is required for further evaluation.

## Discussion

Since the identification of BPV in 1961 and PPV in 1965 [40], [41], new members of the family *Parvoviridae* have emerged, particularly in recent times since the development of random amplification methodologies and high-throughput sequencing [7]–[9], [12], [13], [16], [42]–[44]. This has also resulted in somewhat of a delay in the classification of these newly discovered viruses by ICTV. Among these emerging viruses, PPV4 remains an unclassified vertebrate parvovirus [2]. Interestingly, although PPV4 showed the highest identity and the closest relationship with BPV2 among the members in *Parvoviridae*, it has an additional ORF3, which is similar to the genome organization of Bocaviruses, and because of this, PPV4 was proposed as porcine bocavirus 2 (PoBoV2) [18]. However, this classification remains questionable, as PPV4 shows very limited sequence identity to the known bocaviruses and clusters into a separate clade distant to the clade of the genus *Bocavirus* [6], which was confirmed by the present evolutionary analysis (Figures 4 and 5). The discovery of PPV5 makes this classification even more questionable, since PPV5 has a high genomic identity with PPV4 (64.1–67.3%) without possessing the extra ORF3, while it has the same genomic organization as BPV2, resembling the typical parvovirus genome organization of two major ORFs. Additionally, besides the relative higher homology of PPV4 and PPV5 to BPV2 among the members in the *Parvoviridae* family, the close evolutionary relationship of PPV5 and PPV4 with BPV2 suggests that these three viruses share the same immediate ancestor. Furthermore, the formation of these three viruses into one clade distant from others indicates they could be classified into a separate genus different from those previously defined (Figures 4 and 5).

Recent evidence indicates that the age of the *Parvoviridae* family may exceed 40 to 50 million years [45]. Moreover, besides their long history, the genomes of parvoviruses were revealed to exhibit similar high mutation rates as RNA viruses [46]–[48]. These high mutation rates together with the long history may be the reason for the high diversity and the vast genetic divergence of the family *Parvoviridae*. Based on the present data, PPV5 could be inferred as an intermediate during the evolution of an ancestral PPV which evolved into PPV4, while the co-circulation of PPV4 and PPV5 in pigs might be evidence that the evolution of new PPVs is ongoing.

Parvovirus is known to utilize a rolling hairpin model for replication [24], [49], [50], and in this model the intermediates produced during replication are head-to-head or tail-to-tail concatamers. However, head-to-tail circular genome arrays have been reported in association with AAV infected human tissues and cell lines [25], [26], [51], and recently the head-to-tail or circular genome configuration was revealed in clinical samples infected with human bocavirus 1, 2 and 3 [27], [52], [53]. Moreover, it was discovered that the AAV DNA persisted mainly as circular episomes in human tissues [25], and the head-to-tail circular intermediates were considered to be correlated with the long-term persistence of AAV in tissues or cell lines [26], [51]. Previously, the PPV4 was cloned from a circular or a head-to-tail concatemeric template [6], and it was suggested that PPV4 may be able to establish persistence in its host. In the present study, the genomes of PPV5 were also identified from circular or head-to-tail structures. It is difficult to clarify the mechanism of the formation of these circular or head-to-tail molecules by using PCR amplification with polymerases alone, even at high temperature, as they might result from a PCR reaction on a template with palindromic structure as observed for other parvoviruses [54]. Alternatively, they may also be the products of recombination or a novel feature in the replication cycle or part of a dead-end replication product, as elucidated previously [27]. Regardless of how they were generated, the PPV5 genomes identified in the present study provide new information of the molecular characteristics of newly emerging parvoviruses in pigs, which may help to understand the mechanism of replication of parvoviruses during the evolution process.

Previously, PLA_2_ was reported to play an important role in entering the host cell and being required for infectivity of parvoviruses [37]–[39]. For human parvovirus B19, it has been proposed that PLA_2_ contributes to inflammatory and autoimmune processes and was therefore associated with parvovirus pathogenesis [39]. The PLA_2_ domain was previously identified in PPV1 [37] and in PBoVs [13], [55]. The identification of the putative motif of PLA_2_ in PPV5 may indicate that PPV5 infects cells similar to other paroviruses which possess PLA_2_. However, besides the genome organization, the lack of the PLA_2_ motif in PPV4 indicates yet another substantial difference between PPV4 and PPV5 and perhaps suggests the presence of an alternative domain or other way that could replace the function of PLA_2_ in PPV4 which has yet to be identified.

Our epidemiologic investigation based on U.S. pig samples revealed that based on DNA detection, PPV5 had a slightly higher positive rate than PPV4, specifically, 6.6% versus 4.1% in lung samples and 2.6% versus 1.9% in fecal samples. The overall prevalence of PPV4 DNA in our study was similar to the results reported previously in Hungary and China [19], [22], which may indicate that PPV4 has similar prevalence rates in different geographical locations. Furthermore, the low detection in pre-suckling, suckling and nursery pigs suggests vertical transmission may not be a main route for both PPV4 and PPV5. To the author’s knowledge, serological assays have not been developed for any of the emerging parvoviruses in pigs to date. Based on a small evaluation in this study using serum samples from pigs experimentally infected with PPV1, it appears that antibodies against PPV1 do not react with PPV4 and PPV5 capsid proteins perhaps supporting that development of differential serological tools would be feasible and could be utilized in the future. Further investigations are urgently needed to determine the pathogenic capabilities of both PPV4 and PPV5 under controlled conditions, and more extensive epidemiologic studies including analysis of samples from other geographic regions and countries are necessary to determine the distribution and transmission of PPV4 and PPV5.
